# Null Mutation in PGAP1 Impairing Gpi-Anchor Maturation in Patients with Intellectual Disability and Encephalopathy

**DOI:** 10.1371/journal.pgen.1004320

**Published:** 2014-05-01

**Authors:** Yoshiko Murakami, Hasan Tawamie, Yusuke Maeda, Christian Büttner, Rebecca Buchert, Farah Radwan, Stefanie Schaffer, Heinrich Sticht, Michael Aigner, André Reis, Taroh Kinoshita, Rami Abou Jamra

**Affiliations:** 1 Research Institute for Microbial Diseases and WPI Immunology Frontier Research Center, Osaka University, Suita, Osaka, Japan; 2 Institute of Human Genetics, Friedrich-Alexander-Universität Erlangen-Nürnberg, Erlangen, Germany; 3 Department of Internal Medicine 5, Hematology and Oncology, University of Erlangen-Nürnberg, Erlangen, Germany; 4 Institute of Biochemistry, Friedrich-Alexander-Universität Erlangen-Nürnberg, Erlangen, Germany; Max Planck Institute for Molecular Genetics, Germany

## Abstract

Many eukaryotic cell-surface proteins are anchored to the membrane via glycosylphosphatidylinositol (GPI). There are at least 26 genes involved in biosynthesis and remodeling of GPI anchors. Hypomorphic coding mutations in seven of these genes have been reported to cause decreased expression of GPI anchored proteins (GPI-APs) on the cell surface and to cause autosomal-recessive forms of intellectual disability (ARID). We performed homozygosity mapping and exome sequencing in a family with encephalopathy and non-specific ARID and identified a homozygous 3 bp deletion (p.Leu197del) in the GPI remodeling gene *PGAP1*. *PGAP1* was not described in association with a human phenotype before. PGAP1 is a deacylase that removes an acyl-chain from the inositol of GPI anchors in the endoplasmic reticulum immediately after attachment of GPI to proteins. In silico prediction and molecular modeling strongly suggested a pathogenic effect of the identified deletion. The expression levels of GPI-APs on B lymphoblastoid cells derived from an affected person were normal. However, when those cells were incubated with phosphatidylinositol-specific phospholipase C (PI-PLC), GPI-APs were cleaved and released from B lymphoblastoid cells from healthy individuals whereas GPI-APs on the cells from the affected person were totally resistant. Transfection with wild type *PGAP1* cDNA restored the PI-PLC sensitivity. These results indicate that GPI-APs were expressed with abnormal GPI structure due to a null mutation in the remodeling gene *PGAP1*. Our results add *PGAP1* to the growing list of GPI abnormalities and indicate that not only the cell surface expression levels of GPI-APs but also the fine structure of GPI-anchors is important for the normal neurological development.

## Author Summary

Glycosylphosphatidylinositols (GPI) are glycolipid anchors that anchor various proteins to the cell surface. At least 26 genes are involved in biosynthesis and modification of the GPI anchors. Recently, mutations in eight of those genes have been described. Although those mutations do not fully abolish the functions of encoded enzymes, they lead to a decreased expression of surface GPI-anchored proteins and to different forms of intellectual disability. Here we report a mutation in *PGAP1* that encodes a protein that modifies the GPI anchor. We found that the mutation leads to a full loss of PGAP1 enzyme activity, but that the patient cells still express normal levels of surface GPI-anchored proteins. However, the GPI anchors have an abnormal lipid structure that is resistant to cleavage by phosphatidylinositol-specific phospholipase C. Our results add *PGAP1* to the growing list of GPI abnormalities that cause intellectual disability and indicate that the fine structure of GPI-anchors is also important for a normal neurological development.

## Introduction

Many eukaryotic cell-surface proteins with various functions are anchored to the membrane via glycosylphosphatidylinositol (GPI) [1]–[3]. After biosynthesis in the endoplasmic reticulum (ER), GPI-anchors are transferred to the proteins by the GPI transamidase and the structure of the GPI-anchor is then remodeled, which is critical for sorting, regulating and trafficking of the GPI anchored proteins (GPI-APs) [3]. This remodeling starts in the ER by eliminating the acyl-chain linked to the inositol in the GPI-anchor by PGAP1 [4], then a side-chain of ethanolamine-phosphate on the second mannose of the GPI-anchor is removed by MPPE1 (PGAP5) [5]. GPI-APs are then transported from the ER to the plasma membrane through the Golgi apparatus, where further remodeling by PGAP3 and PGAP2 takes place [6], [7]. Germline mutations in eight genes that are involved in the GPI-anchor biosynthesis and remodeling have been described (Table 1) [8]–[22]. The mutations in all of those, *PIGA*, *PIGL*, *PIGM*, *PIGV*, *PIGN*, *PIGO*, *PIGT* and *PGAP2*, are hypomorphic and lead to partially decreased cell surface expression of various GPI-APs, thus causing a wide phenotypic spectrum ranging from syndromic disorders with various malformations to non-specific forms of intellectual disability. The reported mutations in genes of early steps of the GPI-anchor synthesis such as *PIGA* (MIM 311770), *PIGL* (MIM 605947), and *PIGM* (MIM *610273), or in a gene involved in GPI transfer to proteins such as *PIGT* (MIM *610272) are supposed to result in a degradation of precursor non-GPI-anchored proteins by ER associated degradation, whereas mutations in genes that are involved in later steps of the pathway, such as *PIGV* (MIM *610274), *PIGO* (MIM *614730), and *PGAP2* (MIM *615187) result in partial secretion of non-GPI-anchored proteins such as alkaline phosphatase (in case of *PIGV* or *PIGO* deficiency) [23] or of proteins bearing cleaved GPI-anchor (in case of *PGAP2* deficiency), and are therefore characterized by hyperphosphatasia. Here we report on the identification of a mutation in *PGAP1* that encodes the GPI inositol-deacylase [4]. This leads to a new type of GPI-anchor deficiency manifesting non-specific autosomal recessive intellectual disability (ARID), in which cell surface levels of GPI-APs are not affected whereas the structure of GPI moiety is abnormal.

**Table 1:** Overview of identified mutations in the GPI synthesis pathway and the associated symptoms.

Gene (RefSeq)	Phenotypes	Families	Mutations	References
*PIGA (NM_002641.3)*	Multiple congenital anomalies involving cleft palate, neonatal seizures, central nervous system structural malformations, intellectual disability	3	homo^1^ p.R412*homo p.Leu110delhomo p.Pro93Leu	[9], [10], [11]
*PIGL (NM_004278.3)*	Coloboma, congenital heart disease, ichthyosiform dermatosis, intellectual disability, ear anomalies	5	comp het^2^ p.Leu167Pro & p.Leu92Phefs*15comp het p.Leu167Pro & p.Gln218*homo p.Leu167Procomp het p.Leu167Pro & c.427-1G>A (Splice defect)comp het p.Leu167Pro & p.del17p12-p11.2	[19]
*PIGM (NM_145167.2)*	Portal and hepatic vein thrombosis in early childhood and seizures, no intellectual disability	2	promoter GC-BOX	[8]
*PIGV (NM_017837.3)*	Intellectual disability, characteristic face, seizures, brachytelephalangy, hyperphosphatasia,	14	homo p.Leu302Prohomo p.Ala341Glucomp het p.Ala341Glu & p.Leu59Argcomp het p.Ala341Glu & p.Cys18Tyrcomp het p.Ala341Glu p.Arg469*comp het p.Ala341Glu & p.His385Prohomo p.Gly256Lyscomp het p.Ala341Glu & p.Ala341Valcomp het p.Ala341Glu & p.Cys156Tyrcomp het p.Pro165Gln & p.Cys156Tyr	[13], [14]
*PIGN (NM_012327.5)*	Multiple congenital anomalies, hypotonia, seizures, intellectual disability	2	homo p.Arg709Glncomp het p.Ser270Pro & c.963G>A (Splice defect)	[17], [18]
*PIGO (NM_032634.3)*	Intellectual disability, recognizable facial characteristics, seizures, brachytelephalangy, hyperphosphatasia	4	comp het p.Leu957Phe & c.3069+5G>A(Splice defect)comp het p.Thr788Hisfs*5 & p.Leu957Phecomp het p. Arg119trp & p. Ala834fs*129comp het p.Gln430* & p.Thr130Asn	[12], [15], [16]
*PIGT (NM_015937)*	Intellectual disability, hypotonia, characteristic facial features, seizures, and further skeletal, endocrine, and ophthalmologic findings, hypophosphatasia	1	homo p.Thr183Pro	[20]
*PGAP1 (NM_024989.3)*	Intellectual disability, major and absence epilepsy in 1 sibling, brain atrophy on CT scan	1	homo p.Leu197del	This study
*PGAP2 (NM_001256240.1)*	Severe intellectual disability, absence seizures, hyperphosphatasia	3	homo p.Tyr99Cyshomo p.Arg77Procomp het p.Arg16Trp & p.Thr160IIe	[21], [22]

1: homozygous,

2: compound-heterozygous.

## Results

### Clinical manifestations

**Figure 1:**
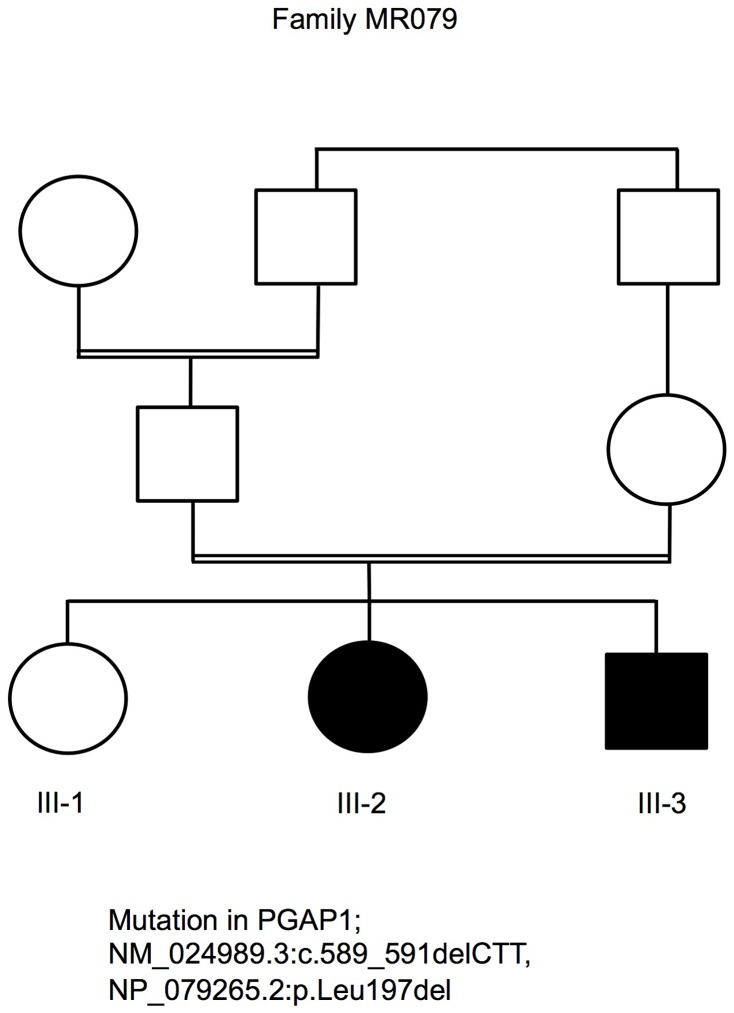
Pedigree of family MR079 and a PGAP1 mutation.

We undertook clinical characterization, mapping [24] and exome sequencing in a large cohort of families with non-specific ARID. We identified the *PGAP1* mutation in the Syrian family MR079. The parents in family MR079 are the first-degree cousins and the family has one healthy girl and two affected children that carry the mutation in a homozygous status. The affected girl (III-2) was 4 years and 5 months old and the affected boy (III-3) was 2 years and 9 months old at the time of examination (Figure 1). Pregnancy, delivery, and birth parameters of both children were unremarkable. In the neonatal period, III-2 was hypotonic and III-3 was a floppy baby. Motor development was delayed; III-2 could sit at age of 18 months and at age of 4^5/12^ years first tried to walk independently. At age of 2^9/12^, III-3 could only roll from back to stomach and back. Both children did not finish potty training and were still partially fed with milk bottles. Both children have a developmental delay and severe intellectual disability with an estimated IQ below 35. III-2 could only babble a few syllables. While III-2 had major and absence epilepsy, III-3 did not yet have seizures. Sleeping patterns of both children were normal. They showed some stereotypic movements such as hitting on their own mouth and some washing movements of the hands. Both children seemed to see and hear properly, but specific tests could not be done. Brain CT scan of III-2 at age of one year revealed pronounced brain atrophy. At the time of examination, III-2 was 96 cm tall (25^th^ percentile) with a head circumference of 46 cm (2 cm below the 5^th^ percentile). III-3 was also of normal height and had a head circumference of 47 cm (1.5 cm below the 5^th^ percentile). Their parents had head circumferences of 52 and 53 cm, also in the lower percentiles. Both children have large ears and a flattened nasal root. G-banding, cytogenetic examination and genome wide copy number variants analyses were unremarkable. We did not have information on the levels of alkaline phosphatase and it was not possible to obtain blood probes retrospectively.

### Exome sequencing revealed a homozygous mutation in *PGAP1*

Autozygosity mapping [24] in family MR079 led to the identification of six candidate regions of a total length of 64 Mb. Subsequently, exome sequencing using DNA from individual III-3 was performed as described in former studies [21], [25] resulting in an average coverage of 53.28. 66% of the target sequences were covered with a depth of at least 20×, and 80.51% were covered with a depth of at least 5×. A total of 42,352 SNVs and 2,529 indels were identified. 342 SNVs and 64 indels were neither annotated, nor reported in 1000Genomes and Exome Variant Server, nor in in-house controls, and may affect the protein sequence (non-synonymous, splicing, or UTR). Of those, only two, in *PGAP1* and *SLC40A*, were located in a candidate region, conserved, and predicted to be pathogenic by *in silico* programs. To exclude further candidate mutations, we repeated the exome sequencing using DNAs of both affected siblings. We enriched the exome using a PCR based targeting method (Ion AmpliSeq Exome Kit) and sequenced on the Ion Proton. The average coverage of III-3 and III-2 was 149.6× and 94.6×, respectively. 91.1% and 85.0% of the target sequences were covered with a depth of at least 20×, 96.3% and 93.4% with a depth of at least 5×, respectively. A total of 49,455 and 47,693 SNVs as well as 3,343 and 3,167 indels were identified. When applying the above mentioned filtering steps, we were by both affected children once again left with the variants in *PGAP1* and *SLC40A*. Since mutations in *SLC40A* cause hemochromatosis of type 4 and have no effect on cognition (MIM 606069) [26], [27], we focused on the variant in *PGAP1*, NM_024989.3:c.589_591delCTT, NP_079265.2:p.Leu197del. Genotyping the variant in *PGAP1* in 372 healthy Syrian adults using Sanger sequencing revealed no further carriers. Taking the minor allele frequency of 0 in the Exome Sequencing Project (ESP) data set and in our control sample of 372 healthy Syrian individuals, it seems that the mutation has prevalence far less than 0.001.

**Figure 2:**
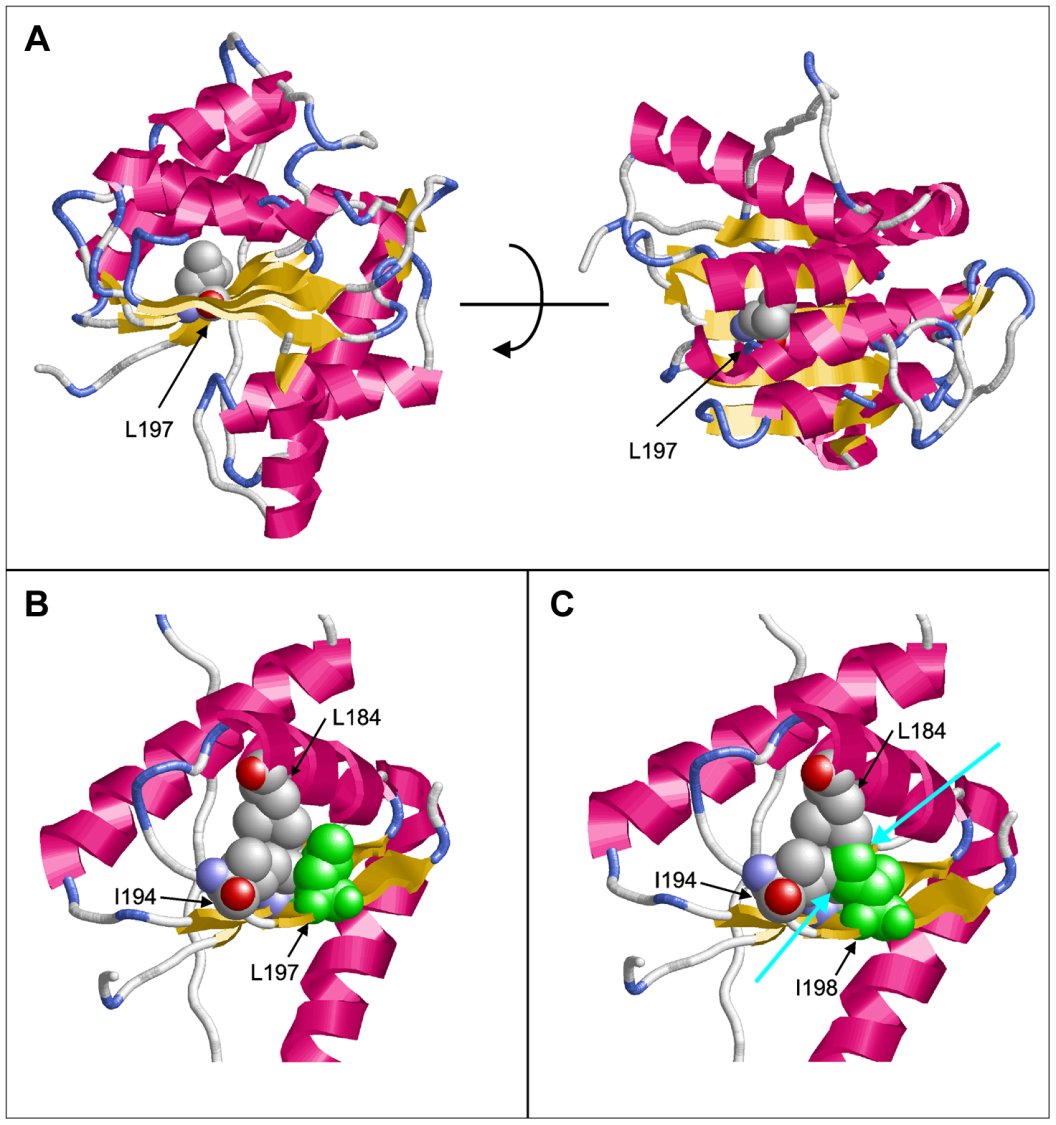
Molecular modeling of PGAP1. (A) Model of PGAP1 highlighting the position of Leu197. The two views differ by a rotation of 90° around the horizontal axis. (B) Interactions of Leu197 (green) with residues Leu184 and Ile194 of the hydrophobic core. (C) Interactions of Ile198 (green) in the Leu197del mutant. Clashes with the adjacent amino acids Leu184 and Ile194 are indicated by cyan arrows. Residues 203–316 are not shown in (B) and (C) for reasons of clarity.

Molecular modeling using the GeneSilico fold recognition metaserver [28] and Modeler9.9 [29] using the closest related hydrolase (PDB code: 3LP5) as template highlighted the detrimental effect of the deletion of leucine 197 on the structure of PGAP1. Leucine 197 is located in the central strand of a β-sheet and is oriented towards the hydrophobic core of the enzyme where it forms multiple stabilizing interactions with the adjacent helices (Figure 2A, B). Deletion of this amino acid would place Ile198 at the position originally occupied by Leu197 (Figure 2C). The Cβ-branched side-chain of isoleucine cannot be accommodated at this sequence position resulting in several clashes with adjacent amino acids (Leu184, Ile194) of the hydrophobic core (Figure 2C). This will disrupt the packing of the hydrophobic core and consequently of the entire β-sheet topology, thus leading to a loss of tertiary structure and enzymatic activity.

We then ran large scale homozygosity mapping using PLINK in our sample of over 100 consanguineous families [24] and over 600 sporadic cases of ID [30] and identified 7 index patients, 2 from consanguineous families with multiple affected children and 5 from outbreed families with single affected patients, that are homozygous at the *PGAP1*. Sequencing all seven individuals using Sanger did not reveal any mutations in *PGAP1*.

We then screened the exome variant server for functional variants in *PGAP1*. 149 variants are reported in this gene, of those 44 were coding or at splice sites. All of those are extremely rare (0.0077%–0.569%, i. e. 1–74 alleles out of ca. 13000 alleles). Based on the conservation of the variants and the prediction of *in silico* programs (Table S1), we roughly estimate that a maximum of 48 individuals may carry a mutation in *PGAP1* (carrier rate of 48/6500 = 0.0073) and that the prevalence of the disease would be about 13 per million. If we take more conservative *in silico* prediction numbers, the prevalence of the disease would be 7 per million inhabitants (Table S1). The two most frequent variants in the ESP data were p.Lys111Glu and p.Gln585Glu and were observed in a heterozygous form 15 and 74 times out of 12992 and 12932 alleles, respectively. Both sites are well conserved in the mammalian. Molecular modeling showed that the most common variant Gln585Glu is located outside of catalytic active domains and it was not possible to make a prediction for this variant. Lys111Glu is at the C terminus of a helix of the deacylase domain. The charging pattern of the helix is highly conserved so that we expect that the change from Lys to Glu would change the charge of the protein and destabilize the helix.

### Flow cytometry of B-lymphoblastoid cell lines

**Figure 3:**
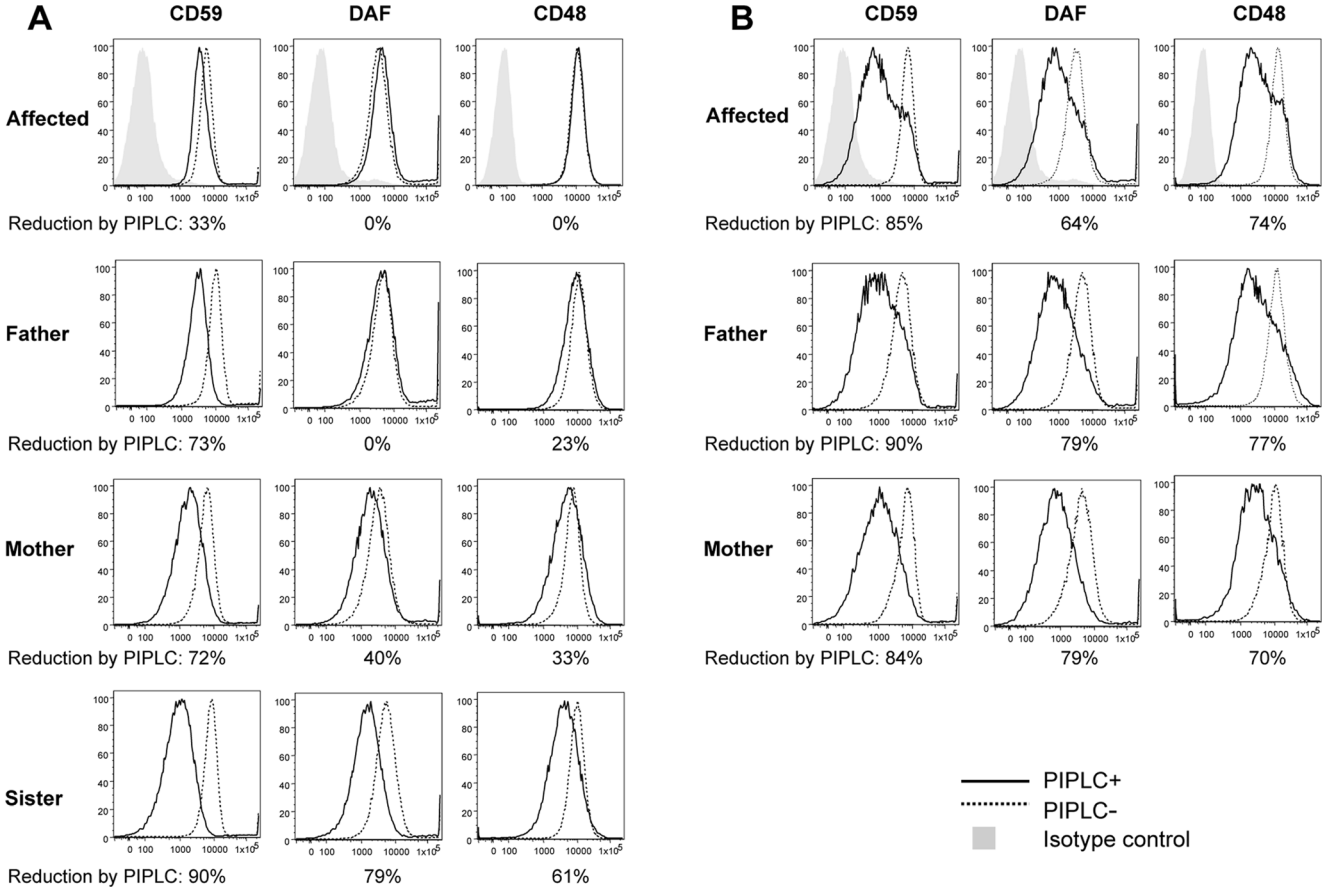
FACS analysis of GPI-APs on LCLs and their PI-PLC sensitivity. (A, B) Cells from one of the affected siblings (III-3) and the parents were transfected with empty pMEoriP vector (A) and pMEoriP-FLAG-humanPGAP1 (B). Cells from the healthy sister were used without transfection. Four days after transfection, cells were treated with (solid lines) or without (dotted lines) 10 unit/ml of PI-PLC for 1.5 h at 37°C, and the surface expression of CD59, DAF and CD48 were assessed by flow cytometry.

To determine effects of p.Leu197del alteration on cellular GPI-APs, we investigated the surface expression of GPI-APs on B-lymphoblastoid cell lines (LCLs) derived from the homozygous individual III-3 (−/−), 2 heterozygous parents (+/−), and the healthy sister (+/+) (Figure 3), as well as 6 healthy volunteers with a confirmed wild type genotype (data not shown). Using flow cytometry analysis, the respective surface expressions of CD59, CD55/DAF, and CD48 were quantified. Surface expression of these GPI-APs on LCLs from an affected person, other family members or healthy volunteers showed no significant difference, indicating that the *PGAP1* mutation did not affect the surface expression levels of various GPI-APs (Figure 3A, dotted lines). The surface expression of the GPI anchor itself was quantified using fluorochrome conjugated aerolysin (FLAER, Pinewood Scientific), a bacterial toxin that specifically binds GPI anchors, and did not show significant differences between the affected individual, the heterozygous individuals, and the controls (data not shown).

### Altered GPI anchors are resistant to PI-PLC cleavage

We then investigated the expected structural abnormality of GPI-anchors by testing sensitivity of GPI-APs to phosphatidylinositol-specific phospholipase C (PI-PLC) [31]. The LCLs were incubated with 10 unit/ml of PI-PLC for 1.5 h at 37°C and the remaining surface GPI-APs were determined by flow cytometry. Of GPI-APs, 61% to 90% were removed from the surface of LCLs of the healthy sister with a homozygous wildtype (Figure 3A, solid line) and healthy control individuals (data not shown). In contrast, no significant or only slight reduction of the surface GPI-APs was seen with LCLs from the affected person (Figure 3A), indicating that almost all GPI-APs on the affected LCLs had abnormal GPI anchors resistant to PI-PLC [4]. This is a strong indication that the p.Leu197del mutation causes null or almost null activity of the PGAP1 enzyme. GPI-APs on LCLs from heterozygous parents were only partially sensitive to PI-PLC (Figure 3A), indicating that the p.Leu197del mutation causes haplo-insufficiency. These defective sensitivities of affected the person's and parents' GPI-APs to PI-PLC were fully restored by transfection of wild-type *PGAP1* cDNA (Figure 3B, solid lines).

**Figure 4:**
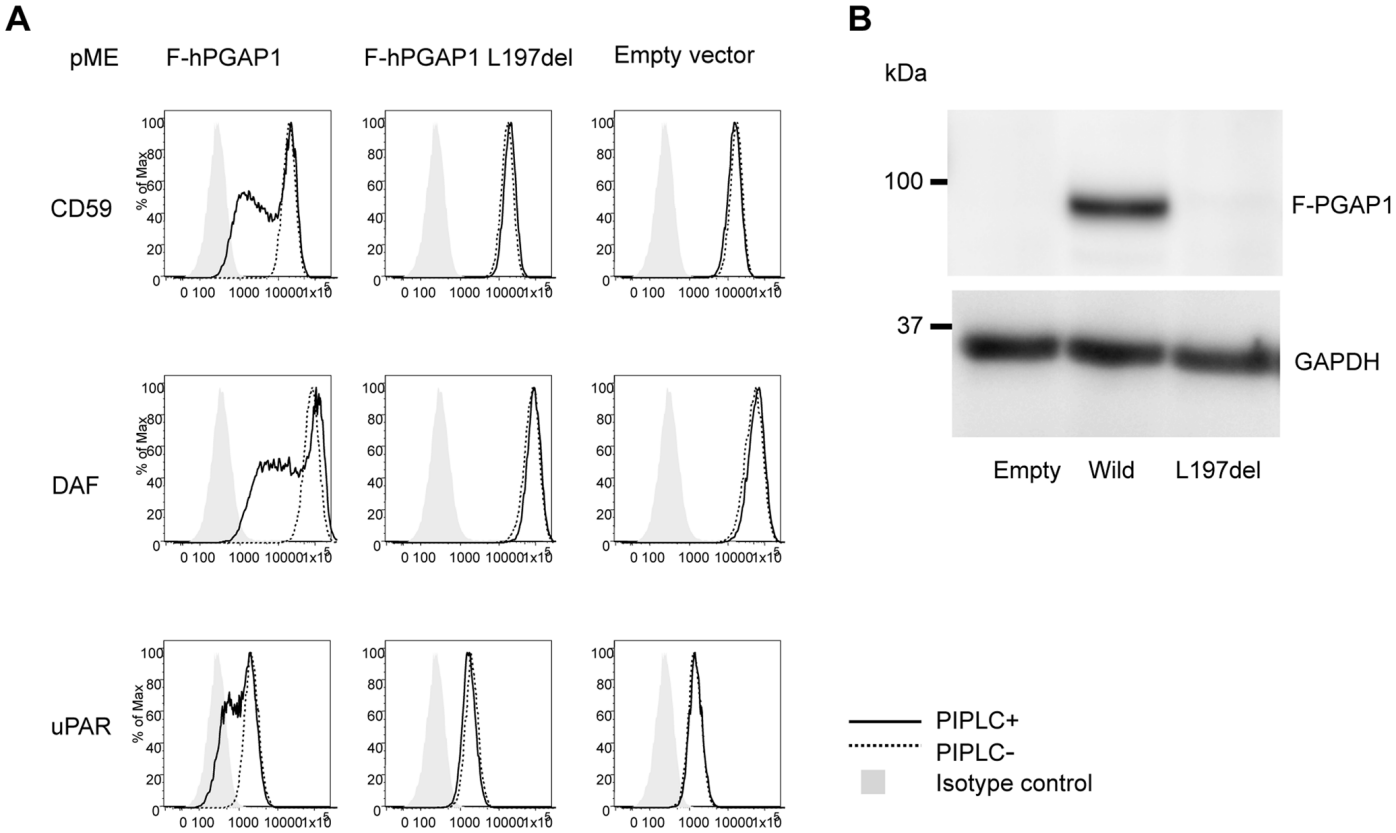
Functional ability of mutant *PGAP1* cDNA. (A) *PGAP1* deficient CHO cell (C10) [4] were transiently transfected with N-terminally-FLAG-tagged wild-type and mutant (L197del) human *PGAP1* driven by a strong promoter SRa, or an empty vector. Four days after transfection, each transfectant was treated with (solid lines) or without (dotted lines) 10 unit/ml of PI-PLC for 1.5 h at 37°C and the surface expression of CD59, DAF and uPAR were assessed by flow cytometry. (B) Two days after transfection of each *PGAP1* construct, lysates were immunoprecipitated with anti-FLAG beads and analyzed by SDS-PAGE/Western blotting. L197del mutant protein was not detected at all.

Finally, the functional effect of the p.Leu197del mutation was tested in the *PGAP1* deficient Chinese hamster ovary (CHO) cell system [4]. GPI-APs expressed on the *PGAP1* deficient CHO cells are resistant to PI-PLC and the activity of *PGAP1* cDNA can be assessed by its ability to make PI-PLC-sensitive GPI-APs after transfection. CHO cells defective for *PGAP1* were transiently transfected with N-terminally-FLAG-tagged wild-type and p.Leu197del mutant human *PGAP1* cDNA in an expression vector with a strong SRα promoter, or an empty vector. Four days after transfection, each transfectant was treated with or without PI-PLC, and the surface expression of CD59, DAF and urokinase plasminogen activator receptor (uPAR) were assessed by flow cytometry. The wild-type *PGAP1* cDNA rescued PI-PLC sensitivity (Figure 4A, left panels). In contrast, the transfection of the mutant p.Leu197del cDNA did not increase the sensitivity to PI-PLC, thus indicating functional loss of the mutant *PGAP1* cDNA (Figure 4A, center panels). To determine PGAP1 protein levels, lysates were prepared two days after transfection, immunoprecipitated with anti-FLAG beads and analyzed by SDS-PAGE/Western blotting. The p.Leu197del mutant protein was not detected at all, indicating that the deletion of Leu197 caused an unstable protein (Figure 4B).

In order to evaluate other known variants in *PGAP1*, we screened the public database of ESP (see above). Of listed variants, we chose the two most frequent variants: rs142320636: c.331A>G (p.Lys111Glu) and rs62185645: c.1753C>G (p.Gln585Glu), and tested the functional effect of these mutations in the *PGAP1* deficient Chinese hamster ovary (CHO) cell system. Transfection of the mutant p.Lys111Glu cDNA did not increase the sensitivity to PI-PLC, indicating functional loss of the mutant *PGAP1* cDNA. Mutant p.Gln585Glu showed an activity comparable to the wild type PGAP1 (Figure S1). Thus, it is possible that homozygosity of p.Lys111Glu leads to ARID.

## Discussion

Eight GPI deficiencies caused by hypomorphic mutations in the coding regions of GPI biosynthesis genes *PIGM*, *PIGA*, *PIGL*, *PIGV*, *PIGN*, *PIGO*, *PIGT*, and *PGAP2* have been reported. Except *PIGM*, all lead to a decreased surface expression of GPI-APs and result in intellectual disability, often associated with epilepsy, distinct facial characteristics, and further organ malformations [9]–[22]. We showed here that complete *PGAP1* deficiency did not affect the surface expression of GPI-APs but expressed structurally abnormal GPI-APs with the acylated inositol.

In previous works, we have reported that *Pgap1* knock-out mice had otocephaly, male infertility, growth retardation, and often died right after birth [32]. Also further two mutant mouse strains, *oto^xray^* (*oto* for otocephaly) [33], [34] and *beaker* [35] were reported to have disrupted *Pgap1*. Both mice strains showed developmental abnormalities of the forebrain; the recessive lethal *oto^xray^* showed a truncation of the forbrain and the *breaker* mutant displayed a holoprosencephaly-like phenotype. Both Wnt signaling and Nodal signaling were reported to be affected in these mutant mice. These data emphasize the importance of *PGAP1* for vital functions and for brain development. It was also indicated that the *Pgap1* mutant mice phenotypes are dependent upon the genetic background since otocephaly and holoprosencephaly are not seen in some mouse strains [34], [35].

Based on our mapping results, exome sequencing data and functional experiments that proved pathogenicity of the mutation, the previous reports on intellectual disability caused by mutations in the GPI synthesis pathway, and the mouse models that clearly show an association between the disruption of *Pgap1* and abnormalities of brain, we consider the deletion of leucine197 to be causative for the severe non-specific autosomal recessive intellectual disability in our examined patients of family MR079. *PGAP1* is the ninth gene of the GPI synthesis pathway that is now associated to a human phenotype (Table 1). Further mutations in *PGPA1* are needed to confirm our findings. Also, describing further patients with different mutations is necessary to delineate the phenotypes of the GPI deficiencies. For example, considering the defect in the modification of the GPI anchors, the alkaline phosphatase would not be elevated in patients with *PGAP1* mutations, but this needs to be confirmed.

In conclusion, null mutations in *PGAP1* lead to severe intellectual disability and encephalopathy with no obvious malformations; we add *PGAP1* to the growing number of genes involved in GPI-anchor deficiencies with human phenotypes. *PGAP1* deficiency causes a defect in the ER part of the GPI-AP biosynthesis that involves the remodeling of the anchors after attachment to proteins, and it leads to normal protein expression on the cell surface but to abnormal anchor structure.

## Materials and Methods

The study was approved by the Ethic Committees of the Universities of Bonn and of Erlangen-Nürnberg in Germany, and Osaka University in Japan. Informed consent of all examined persons or of their guardians was obtained.

### Mapping and exome sequencing

Genomic DNA was extracted from EDTA blood probes by standard methods and genotyped with the Affymetrix Mapping array 6.0 (Affymetrix, Santa Clara, CA, USA). Analysis did not reveal pathogenic deletions or duplications. Mendelian segregation was calculated using PedCheck software and was confirmed in all instances. Autozygosity mapping was performed using HomozygosityMapper [36]. DNA from individual III-3 was enriched using the SureSelect Human All Exon Kit, which targets approximately 50 Mb of human genome (Agilent, Santa Clara, Ca, USA) and paired-end sequenced on a SOLiD 5500 xl instrument (Life Sciences, Carlsbad, CA, U.S.A.). Image analysis and base calling was performed using the SOLiD instrument control software with default parameters. Read alignment was performed with LifeScope 2.5 using the default parameters with human genome assembly hg19 (GRCh37) as reference. Single-nucleotide variants and small insertions and deletions (indels) were detected using LifeScope, GATK 2 and samtools/bcftools [37], [38]. To replicate the results, DNA from individuals III-2 and III-3 was amplified using the Ion AmpliSeq Exome Kit (Life Technologies, Carlsbad, CA, U.S.A.) which targets approximately 58 Mb of the human genome. After quality control on the Bioanalyzer High Sensitivity Chip (Agilent, Santa Clara, Ca, USA) and emulsion PCR (Ion PI Template OT2 200 Kit v3, Life Technologies, Carlsbad, CA, U.S.A.) the samples were sequenced on a Proton PI chip Version 2 (Life Technologies, Carlsbad, CA, U.S.A.). Base calling, pre-processing of the reads, short read alignment and variant calling was performed using the Torrent Suite including the Torrent Variant Caller (TVC, Version 4.0) with default parameters recommended for the Ampliseq Exome panel (low stringency calling of germline variants, Version September 2013). Variant annotation was performed using Annovar, integrating data from a variety of public databases [39], [40]. Additionally, variants were compared to an in-house database containing more than 350 sequenced exomes to identify further common variants which are not present in public databases. Finally, the variants were validated by PCR and Sanger sequencing according to the standard protocols to exclude technical artifacts and to test for segregation.

### PI-PLC treatment and FACS analysis

Heparin blood samples were collected from one affected and from all unaffected siblings and parents. Lymphoblastoid Cell lines (LCLs) were generated and cultured in RPMI 1640 (Gibco, Life technologies, Darmstadt, Germany) that is supplemented with 10% FCS (PAA Biotech, Cölbe, Germany) and different other supplements. LCLs from one of the affected siblings (III-3) and the parents were transfected with empty pMEoriP vector or pMEoriP-FLAG-humanPGAP1. Cells from healthy sister were used without transfection. Cells (5×10^6^) were suspended in 0.8 ml of Opti-MEM and electroporated with 20 µg each of the plasmids at 260 V and 960 µF using a Gene Pulser (Bio Rad, Hercules, CA). Four days after transfection, cells were treated with or without 10 unit/ml of PI-PLC (Molecular probes, Eugene, OR) for 1.5 h at 37°C. Surface expression of GPI-APs was determined by staining cells with mouse anti-human CD59 (5H8), -human DAF (IA10), -human CD48 (BJ40) antibodies and each isotype IgG followed by a PE-conjugated anti-mouse IgG antibody (BJ40, mouse IgG1 and IgG2a, and secondary antibody were purchased from BD Biosciences, Franklin Lakes, NJ) and analyzed by flow cytometer (Cant II; BD Biosciences) using Flowjo software (Tommy Digital Inc., Tokyo, Japan).

### Functional analysis using CHO cells

pMEFLAG-hPGAP1 mutant (L197del) bearing patient's mutation was generated by site directed mutagenesis. *PGAP1* deficient CHO cell (C10) [4] were transiently transfected with wild type or mutant pMEFLAG-hPGAP1 by electroporation. Cells (10^7^) were suspended in 0.4 ml of Opti-MEM and electroporated with 20 µg each of the plasmids at 260 V and 960 µF using a Gene Pulser. Four days after transfection, cells were treated with or without 10 unit/ml of PI-PLC for 1.5 h at 37°C. Surface expression of GPI-APs was determined by staining cells with mouse anti-human CD59 (5H8), -human DAF (IA10), -hamster uPAR (5D6) antibodies and each isotype IgG, followed by a PE-conjugated anti-mouse IgG antibody and analyzed by flow cytometer using Flowjo software. Two days after transfection of each *PGAP1* construct, lysates were immunoprecipitated with anti-FLAG beads and analyzed by SDS-PAGE/Western blotting.

### Web resources

1000Genomes, http://www.1000genomes.org/

ABI, L.T. (2012). LifeScope.: http://www.lifetechnologies.com/lifescope.

ANNOVAR: http://www.openbioinformatics.org/annovar/

GeneTalk: http://www.gene-talk.de

BWA, Burrows-Wheeler Aligner; http://bio-bwa.sourceforge.net/

dbSNP, NCBI: http://www.ncbi.nlm.nih.gov/snp/

GATK 2, Genome Analysis Toolkit: http://www.broadinstitute.org/gatk/index.php

Kyoto Encyclopedia of Genes and Genomes, KEGG, http://www.genome.jp/kegg/

MutationTaster: http://www.mutationtaster.org/ELAND, alignment algorithm, Illumina.com

NHLBI Exome Sequencing Project (ESP): http://evs.gs.washington.edu/EVS/

Online Mendelian Inheritance in Man (OMIM): http://www.omim.org

PolyPhen2: http://genetics.bwh.harvard.edu/pph2/

SIFT: http://sift.jcvi.org/

UCSC Genome Browser: www.genome.ucsc.edu

## Supporting Information

Figure S1 Functional ability of mutant *PGAP1* cDNA. *PGAP1* deficient CHO cell (C10) [4] were transiently transfected with N-terminally-FLAG-tagged wild-type and mutant (Lys111Glu, Gln585Glu) human *PGAP1* or an empty vector driven by a strong promoter SRα (pME) or a weak promoter containing only TATA box (pTal). Four days after transfection, each transfectant was treated with (solid lines) or without (dotted lines) 10 unit/ml of PI-PLC for 1.5 h at 37°C and the surface expression of CD59 was assessed by flow cytometry.
(JPG)

Table S1 A list of all ESP database variants with a possible pathogenic effect (i. e. coding or at splice sites). We undertook further in silico analyses using MutationTaster and SIFT and presented in the last two columns estimations about the pathogenicity of the variants. Taking those estimations and the number of identified alleles, one can estimate the prevalence of the disease in the population to be between 7 and 13 per million.
(PDF)
