# Supplementary figures and images for: Null Mutation in PGAP1 Impairing Gpi-Anchor Maturation in Patients with Intellectual Disability and Encephalopathy
Source: PLoS Genet. 2014 May 1;10(5):e1004320. doi: 10.1371/journal.pgen.1004320 (PMC4006728; doi:10.1371/journal.pgen.1004320)

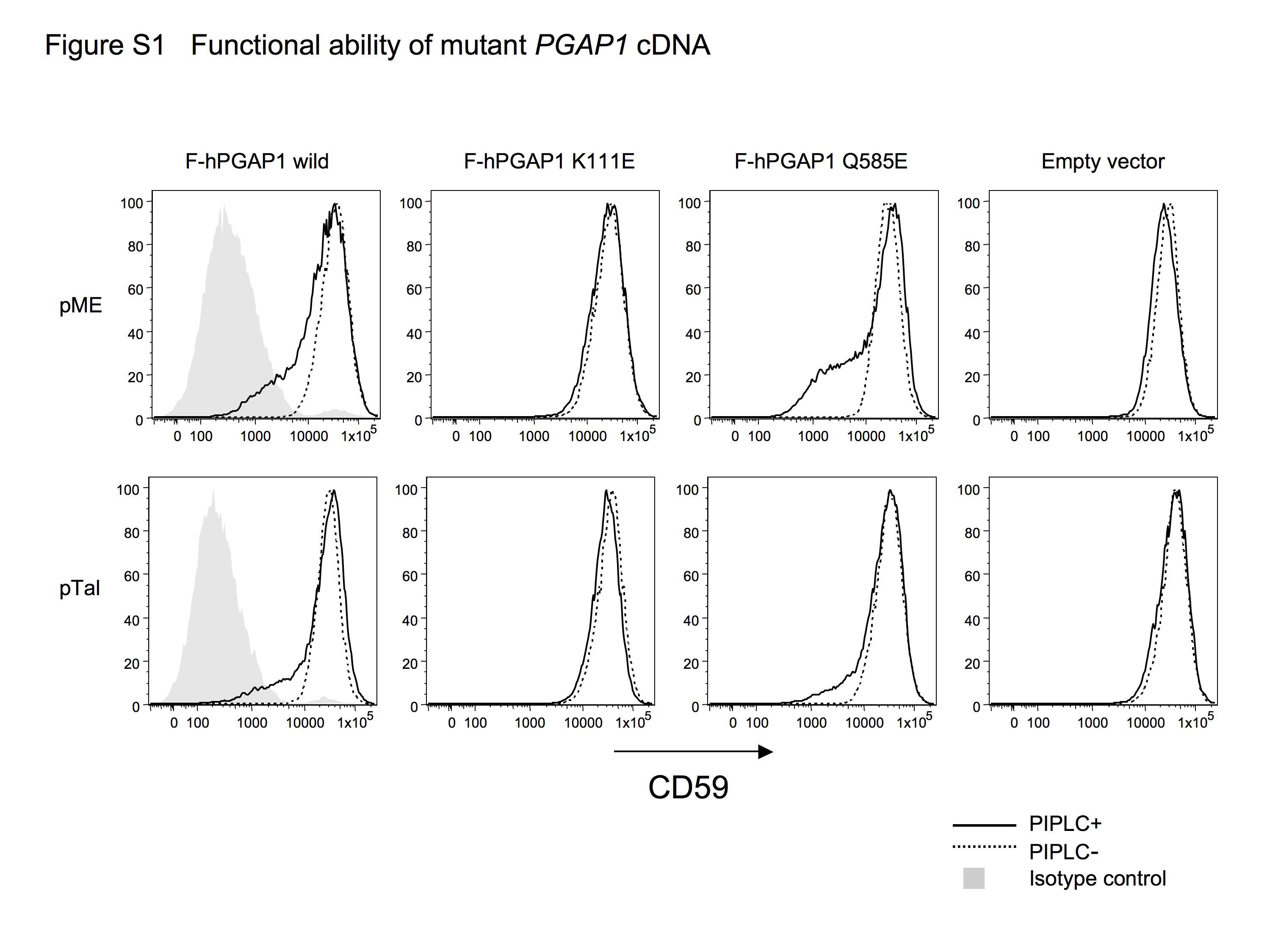

Supplement: Figure S1 — Functional ability of mutant PGAP1 cDNA. PGAP1 deficient CHO cell (C10) [4] were transiently transfected with N-terminally-FLAG-tagged wild-type and mutant (Lys111Glu, Gln585Glu) human PGAP1 or an empty vector driven by a strong promoter SRα (pME) or a weak promoter containing only TATA box (pTal). Four days after transfection, each transfectant was treated with (solid lines) or without (dotted lines) 10 unit/ml of PI-PLC for 1.5 h at 37°C and the surface expression of CD59 was assessed by flow cytometry. (JPG) [file pgen.1004320.s001.jpg]
