# Supplementary material for: Null Mutation in PGAP1 Impairing Gpi-Anchor Maturation in Patients with Intellectual Disability and Encephalopathy
Source: PLoS Genet. 2014 May 1;10(5):e1004320. doi: 10.1371/journal.pgen.1004320 (PMC4006728; doi:10.1371/journal.pgen.1004320)
Supplement: Table S1 — A list of all ESP database variants with a possible pathogenic effect (i. e. coding or at splice sites). We undertook further in silico analyses using MutationTaster and SIFT and presented in the last two columns estimations about the pathogenicity of the variants. Taking those estimations and the number of identified alleles, one can estimate the prevalence of the disease in the population to be between 7 and 13 per million. (PDF) [file pgen.1004320.s002.pdf]

| | | | Genotype count | | | | | | Conservation | | in silico prediction (score) | | | Evaluation* | |
|---|---|---|---|---|---|---|---|---|---|---|---|---|---|---|---|
| Position | rsID | Alleles | AA | AB | BB | FunctionGVS | Protein | cDNA | PhastCons | GERP | MutationTaster | Polyphen2 | SIFT | Possibly | Probably |
| 2:197706050 | rs142041934 | C>A | AA=0 | AC=2 | CC=6501 | missense | p.(G893C) | c.2677G>T | 0.979 | 4.01 | B(0,850) | D(0.982) | B(0.12) | | |
| 2:197706080 | rs139107571 | T>C | CC=0 | CT=5 | TT=6498 | missense | p.(T883A) | c.2647A>G | 0.094 | 0.79 | B(0,999) | B(0.0) | B(1) | | |
| 2:197706085 | rs375115788 | T>C | CC=0 | CT=2 | TT=6501 | missense | p.(K881R) | c.2642A>G | 1.0 | 3.73 | B(0,83) | D(0.974) | B(0.55) | | |
| 2:197708631 | rs377442526 | A>G | GG=0 | GA=1 | AA=6502 | missense | p.(Y836H) | c.2506T>C | 0.829 | 5.18 | D(0996) | D(0.999) | B(0.56) | Mutation | |
| 2:197708736 | rs143253730 | G>A | AA=0 | AG=1 | GG=6502 | missense | p.(H801Y) | c.2401C>T | 0.003 | 3.71 | B(0,999) | B(0.106) | B(1) | | |
| 2:197708762 | rs36086547 | T>C | CC=0 | CT=7 | TT=6496 | missense | p.(N792S) | c.2375A>G | 0.007 | -5.03 | B(0,999) | B(0.0) | B(1) | | |
| 2:197708776 | - | ACTT>A | A1A1=0 | A1R=1 | RR=6259 | coding | p.(R786_S787delinsS) | c.2358_2360del3 | 1.0 | 4.62 | D(0,735) | - | - | Mutation | Mutation |
| 2:197708788 | - | GTGTT>G | A1A1=0 | A1R=1 | RR=6259 | frameshift | p.(K782Tfs*37) | c.2345_2348del4 | 0.94 | 2.78 | D(1) | - | - | Mutation | Mutation |
| 2:197709252 | rs371515536 | G>A | AA=0 | AG=1 | GG=6453 | missense | p.(P778L) | c.2333C>T | 0.998 | 3.85 | D(0,99) | B(0.001) | B(0.36) | | |
| 2:197709298 | rs143960563 | C>T | TT=0 | TC=10 | CC=6448 | missense near-splice | p.(V763I) | c.2287G>A | 0.995 | 0.88 | B(0,63) | B(0.0) | B(0.43); | | |
| 2:197710638 | rs140021567 | T>C | CC=0 | CT=1 | TT=6501 | missense | p.(I752V) | c.2254A>G | 1.0 | 4.23 | B(0,96) | B(0.11) | B(0.14) | | |
| 2:197710708 | rs149424856 | T>C | CC=0 | CT=2 | TT=6501 | missense | p.(I728M) | c.2184A>G | 0.066 | 0.41 | B(0,999) | B(0.003) | B(0.23) | | |
| 2:197711793 | rs370811550 | T>C | CC=0 | CT=1 | TT=6502 | missense | p.(Y695C) | c.2084A>G | 1.0 | 4.82 | D(0,998) | D(1.0) | D(0.01) | Mutation | Mutation |
| 2:197711869 | rs145715800 | C>T | TT=0 | TC=1 | CC=6502 | missense | p.(V670I) | c.2008G>A | 0.961 | 3.78 | B(0,999) | B(0.0) | B(1); | | |
| 2:197711898 | rs143904806 | T>C | CC=0 | CT=1 | TT=6502 | missense | p.(D660G) | c.1979A>G | 0.914 | 4.93 | D(0,973) | D(0.996) | B(0.47) | Mutation | |
| 2:197711924 | rs148349860 | C>T | TT=0 | TC=1 | CC=6502 | coding-syn near-splice | p.(G651=) | c.1953G>A | 0.998 | -1.5 | D(1) | - | - | | |
| 2:197712747 | rs150893861 | A>G | GG=0 | GA=7 | AA=6486 | missense | p.(Y626H) | c.1876T>C | 0.873 | 4.09 | B(0,999) | D(0.978) | D(0.04) | Mutation | |
| 2:197729765 | rs139880907 | C>T | TT=0 | TC=1 | CC=6498 | missense | p.(V603I) | c.1807G>A | 0.007 | -1.71 | B(0,999) | B(0.0) | B(0.39) | | |
| 2:197729788 | rs372173608 | C>A | AA=0 | AC=1 | CC=6497 | missense | p.(G595V) | c.1784G>T | 1.0 | 5.0 | D(0,999) | D(0.987) | D(0.04) | Mutation | |
| 2:197735679 | rs62185645 | G>C | CC=0 | CG=74 | GG=6429 | missense | p.(Q585E) | c.1753C>G | 1.0 | 4.63 | D(0,513) | B(0.259) | B(0.53); | | |
| 2:197737163 | rs376512426 | A>G | GG=0 | GA=1 | AA=6502 | splice-5 | NA | c.1728+2T>C | 0.99 | 5.15 | - | - | - | | |
| 2:197737266 | rs138897526 | A>T | TT=0 | TA=1 | AA=6502 | missense | p.(S543T) | c.1627T>A | 0.998 | 3.84 | D(0,88) | B(0.02) | B(0.35) | | |
| 2:197737745 | rs201002323 | T>C | CC=0 | CT=1 | TT=6364 | missense | p.(I520V) | c.1558A>G | 1.0 | 3.68 | B(0,998) | B(0.114) | B(0.24) | | |
| 2:197738413 | rs201928408 | C>T | TT=0 | TC=1 | CC=6489 | missense | p.(G499E) | c.1496G>A | 1.0 | 4.01 | B(0,994) | D(0.845) | B(0.37) | | |
| 2:197738434 | rs199809707 | T>C | CC=0 | CT=1 | TT=6493 | missense | p.(N492S) | c.1475A>G | 1.0 | 3.9 | B(0,989) | B(0.199) | B(0.65) | | |
| 2:197740500 | rs143038880 | G>A | AA=0 | AG=2 | GG=6490 | stop-gained | p.(Q466*) | c.1396C>T | 1.0 | 5.32 | - | - | - | Mutation | Mutation |
| 2:197763089 | rs377188685 | G>C | CC=0 | CG=1 | GG=6502 | stop-gained | p.(S271*) | c.812C>G | 1.0 | 5.21 | - | - | - | Mutation | Mutation |
| 2:197767347 | rs374565749 | G>A | AA=0 | AG=1 | GG=6502 | missense | p.(P257S) | c.769C>T | 1.0 | 4.14 | D(0,970) | D(0.885) | B(0.46) | Mutation | |
| 2:197767371 | rs141210056 | C>T | TT=0 | TC=2 | CC=6501 | missense | p.(V249I) | c.745G>A | 1.0 | 4.95 | D(0,999) | D(1.0) | D(0) | Mutation | Mutation |
| 2:197767424 | rs368553507 | C>T | TT=0 | TC=1 | CC=6502 | missense | p.(R231Q) | c.692G>A | 0.826 | 1.87 | B(0,998) | B(0.054) | B(0.85) | | |
| 2:197777673 | rs371517233 | T>C | CC=0 | CT=2 | TT=6501 | missense | p.(I194M) | c.582A>G | 1.0 | 1.59 | D(0,941) | D(0.934) | D(0.01) | Mutation | |
| 2:197777699 | rs370198742 | G>T | TT=0 | TG=1 | GG=6502 | missense | p.(L186M) | c.556C>A | 0.865 | -5.72 | B(0,968) | D(0.993) | D(0.01) | | |
| 2:197777744 | rs372875380 | T>C | CC=0 | CT=1 | TT=6502 | missense | p.(I171V) | c.511A>G | 1.0 | 1.61 | B(0,555) | B(0.0) | D(0.01) | | |
| 2:197777755 | rs144512765 | C>T | TT=0 | TC=1 | CC=6502 | missense | p.(S167N) | c.500G>A | 1.0 | 5.25 | D(0,999) | D(0.999) | D(0.02) | Mutation | Mutation |
| 2:197781253 | rs374426820 | G>T | TT=0 | TG=1 | GG=6502 | missense | p.(D122E) | c.366C>A | 1.0 | 4.69 | D(0,747) | D(0.987) | B(0.09); | Mutation | |
| 2:197781288 | rs142320636 | T>C | CC=0 | CT=15 | TT=6488 | missense | p.(K111E) | c.331A>G | 1.0 | 5.58 | D(0,999) | D(0.999) | B(0.1) | Mutation | Mutation |
| 2:197781309 | rs200242648 | T>C | CC=0 | CT=2 | TT=6501 | missense | p.(I104V) | c.310A>G | 0.881 | -7.56 | B(0,991) | B(0.0) | D(0) | | |
| 2:197784745 | rs151266857 | C>G | GG=0 | GC=1 | CC=6502 | missense | p.(G93R) | c.277G>C | 1.0 | 5.25 | D(0,999) | D(1.0) | - | Mutation | Mutation |
| 2:197784771 | rs114297432 | G>A | AA=0 | AG=4 | GG=6499 | missense | p.(T84M) | c.251C>T | 0.988 | 5.25 | D(0,999) | D(1.0) | - | Mutation | Mutation |
| 2:197784835 | rs201585822 | C>T | TT=0 | TC=1 | CC=6502 | missense | p.(A63T) | c.187G>A | 0.988 | 1.34 | D(0,899) | B(0.231) | - | | |
| 2:197791215 | rs145141597 | C>G | GG=0 | GC=1 | CC=6502 | missense | p.(M42I) | c.126G>C | 1.0 | 4.16 | D(0,999) | D(0.998) | - | Mutation | Mutation |
| 2:197791237 | rs149238260 | T>C | CC=0 | CT=4 | TT=6499 | missense | p.(N35S) | c.104A>G | 1.0 | 4.16 | D(0,999) | D(0.993) | - | Mutation | Mutation |
| 2:197791297 | rs373419311 | A>G | GG=0 | GA=1 | AA=6502 | missense | p.(V15A) | c.44T>C | 0.977 | 0.78 | B(0,999) | B(0.0) | - | | |
| 2:197791310 | rs375123215 | G>C | CC=0 | CG=1 | GG=6502 | missense | p.(L11V) | c.31C>G | 1.0 | 0.76 | B(0,993) | B(0.004) | - | | |

* our estimation if a variant is a mutation based on conservation and in silico prediction
